# Sexual Practices of Female Sex Workers Who Inject Drugs in Osogbo, Nigeria

**DOI:** 10.1155/2014/103128

**Published:** 2014-11-06

**Authors:** Ademola Lukman Adelekan, Philomena Imade Omoregie, Elizabeth Ronami Edoni

**Affiliations:** ^1^Department of Research and Reproductive Health, Public Health Promotion Alliance, Osogbo 3166, Nigeria; ^2^Department of Health Promotion and Education, Faculty of Public Health, University of Ibadan, Ibadan 2000005, Nigeria; ^3^Department of Community Health Nursing, Niger-Delta University, Wilberforce Island 569108, Nigeria

## Abstract

Female sex workers (FSWs) who inject drugs have higher risks of HIV infection due to injection drug use and the array of sexual practices employed. This study, therefore, is designed to determine sexual practices of FSWs who inject drug in Osogbo, Nigeria. This study was a cross-sectional descriptive mixed-methods design. Twenty-seven FSWs who inject drug were selected from 11 brothels by snowball sampling and interviewed with a semistructured questionnaire and in-depth interview guide. The mean age of respondents was 26.2 ± 7.5. Many of the respondents were aware of the magnitude of HIV and some were sex workers first before turning to be drug users. Some of the respondents had ever tested for HIV and few had ever been treated for STI more than once. Some respondents were willing to have male clients who do not wear a condom in exchange for accepting more money in return. Many of the respondents reported use of condom, regular talking of herbs, and good personal hygiene as ways of protecting themselves from HIV. Respondents have relatively high numbers of sexual partners. Involving sex workers directly in HIV prevention campaigns will encourage them to look after their health and to access services that could help them.

## 1. Introduction

HIV/AIDS is spreading rapidly in the society and the major cause is attributed to commercial sex workers sexual behaviour [1]. Higher risk of HIV transmission is determined by frequent partner change or higher number of partners; lack of, low level, or inconsistent condom use; unprotected anal intercourse; and presence of certain types of STIs, especially genital ulcerative disease, as cofactors [2]. HIV prevalence among sex workers in Nigeria has remained high over the past decade [3]. HIV prevalence among sex workers in 2007 was over 30%, and in some cities like Kano and Abuja, 50% of all brothel-based sex workers are HIV-infected [3]. Thus, while HIV prevalence among the general population in Nigeria has been declining from its peak of 5.8% in 2001 to 4.1% in 2011 [4] prevalence among brothel-based sex workers has shown no sign of declining [5].

In a recent survey among most-at-risk populations in six states in Nigeria, over half of sex workers did not consider themselves at risk of HIV infection [3]. In Lagos state, with the highest concentration of sex workers in Nigeria, only 16% of brothel-based sex workers felt they were at risk, even though each has on average 34 clients per week [3]. Despite their high-risk sexual activity, many sex workers perceive their risk of HIV infection to be low [6].

As in most regions globally, many African individuals support themselves and their dependants through sex work, primarily women [7]. Between 1% and 4% of women in several West African capital cities are estimated to be sex workers, with even higher proportions in areas were transport and employment networks increase the demand for paid sex [8, 9]. Estimated 37% of sub-Saharan female sex workers (FSWs) live with HIV [10]. The risks of HIV transmission experienced by sex workers who inject drugs are related to their individual exposure to factors such as unprotected sex with multiple partners or sharing injecting equipment while injecting drugs [2]. There is evidence from many countries that sex work rates are much higher among female than among male injecting drug users and that female injectors are more likely to have less control over sharing of injecting equipment in a group injection or even with a sexual partner and are more likely to inject after male injecting drug users, thereby increasing their risk for acquiring HIV [11].

Osun state has consistently experienced fluctuating HIV prevalence since 2001. The HIV prevalence decreased from 4.3% in 2001 to 1.2% in 2003 and again increased to 2.0% in 2005 and decreased to 1.2% in 2008 [12, 13]. Factors driving the epidemic in Osun State include poverty, multiple sex partners, marital infidelity, high unprotected sexual activities among youths, ignorance, low risk assessment, negative cultural activities such as female circumcision, and migration of people from the high prevalence states that share borders with Osun [13]. The sexual behaviour of sex workers according to [14] has been identified as the likely central problem of the transmission of HIV/AIDS because the business may expose them to the danger of contracting HIV infections if they are ignorant of the use of condom as a protective measure against HIV virus as they engage in sex with multisexual partners in respect of their HIV/AIDS status [14]. This study therefore investigates sexual practices of female sex workers who inject drugs in Osogbo, Nigeria.

## 2. Materials and Methods

### 2.1. Study Design and Scope

This study was a cross-sectional descriptive mixed-methods design. The scope of the study was delimited to sexual behaviour, drug use, awareness of HIV magnitude, and HIV counseling and testing (HCT), condom use, and sexually transmitted infection (STI) challenges among FSWs.

### 2.2. Study Area

Osogbo is a city in Nigeria, the capital of Osun State. Osogbo lies on the railway line from Lagos to Kano and is the trade centre for a farming region. Most of the population are members of the Yoruba ethnic group. Osogbo is the venue of the annual Osun-Osogbo festival along the River Osun. The festival is centered around the sacred grove of the river goddess Ọsun, which is a UNESCO World Heritage Site. The population of the state according to the 2006 National Population Census figures is 3,423,535, including 1,740,619 (50.8%) men and 1,682,916 (49.2%) women [13]. Osun state consists chiefly of the Yoruba people, but with variations in dialects; approximately half of the residents are Muslim, with several mosques in major towns such as Iwo, Ede, Osogbo, Ikire, and Ejigbo.

### 2.3. Study Population

The study population was brothel-based FSWs who inject drugs in Osogbo, Osun State, Nigeria.

### 2.4. Sampling Technique

The recruitment of participants was done using purposive sampling. Snowball technique was used in selecting a total of 27 FSWs who inject drugs from 11 brothels (Urban = 6 brothels; semiurban = 5 brothels) in Osogbo. This implies that the participants were selected because they are sex workers and also injecting drug users.

### 2.5. Study Instruments

In-depth interview guide and semistructured questionnaire were developed by the researchers based on the literature reviewed together with input from Health Promotion Specialists at the University of Ibadan, Nigeria. The questionnaire was used to document female sex workers sociodemographic characteristics and sexual behaviour, while in-depth interview questions focused on drug use, awareness of HIV magnitude and HIV counseling and testing (HCT), condom use, and STI challenges.

### 2.6. Data Collection Process

Two of the researchers moderated all the interviews, while note-taking was done by other research members to complement the audio recording by the moderator. Questionnaire was self-administered before the interview and IDIs were conducted in a quiet space within their brothels. Each interview lasted about 30–45 minutesand the discussions were audiotaped with the consent of the participants.

### 2.7. Validity and Reliability of Instruments

The study instruments were reviewed by health promotion experts at the Faculty of Public Health, University of Ibadan, and were also pretested among FSWs who are also injecting drug users in Ibadan which has the same characteristics with the study area to determine how effective the developed instrument would be in collecting appropriate data relevant to the research objectives. A total of four IDIs and questionnaire each were conducted during the pretest and they were revised based on the issues identified by the team.

### 2.8. Data Management and Analysis

The quantitative data collected was collated, screened, and entered into computer. The Statistical Package for Social Science (SPSS) was used to generate frequency tables. For the qualitative data, the analysis was done by reading through the transcribed interviews and listening to the audio records in order to get all major discussions during the interviews and the data were manually analyzed using content analysis technique.

### 2.9. Ethical Consideration

Approval for the study was sought from the Osun State Ministry of Health. Written consent of all the participants was obtained before the interview by giving them informed consent form to fill according to their ability to read and write after explaining the objectives of the study to them. Participation in the study was voluntary and there was no criticism of participants who refused to participate or wish to withdraw from the study. No identifier like participants' name or address was recorded so as to keep the information given by each participant confidential.

## 3. Results

### 3.1. Respondents' Sociodemographic Characteristics

The age of respondents ranged from 21 to 38 with mean age of 26.2 ± 7.5. Many (63.0%) of the respondents were single, married (7.4%), and separated (29.6%). Majority of the respondents (74.1%) had at least secondary school certificate. The respondents have been in the business for an average of 5.3 years (Table 1).

### 3.2. Respondents' Sexual Behaviour

**Figure 1:**
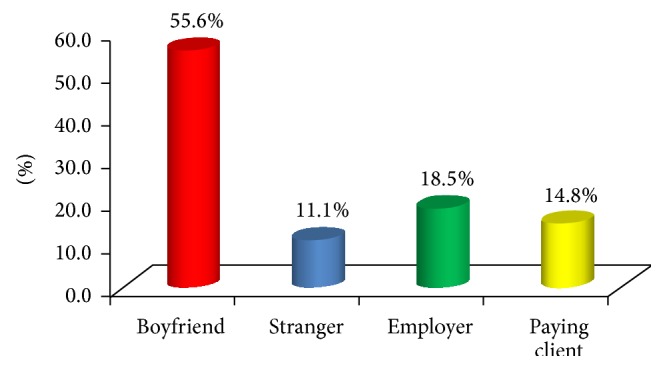
Respondents' first sexual partner.

**Figure 2:**
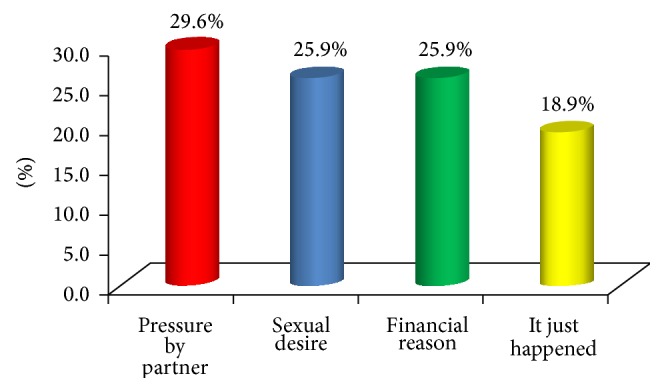
Respondents' reason for first intercourse.

**Figure 3:**
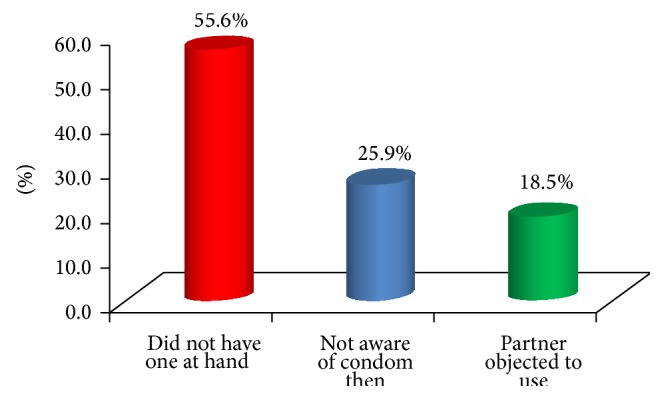
Respondents' reasons for not using condom at first intercourse.

The age of respondents at first intercourse ranged from 13 to 19 with mean age of 15.7 ± 3.2. More than half of the respondents (55.6%) reported boyfriend, as first sexual partner, and employer (18.5%) (Figure 1). Reasons adduced by respondents for first sexual intercourse included pressure by partner (29.6%) and financial reason (25.9%) (Figure 2). All (100.0%) the respondents' reported not using a condom at their first intercourse and their reasons included that they did not have one at hand (55.6%) (Figure 3). Majority of the respondents reported having a daily average of 4 clients and usually spending at least 30 minutes with the client. The majority (88.9%) of the respondents were current family planning users, and intrauterine device (IUD) (54.2%) was a major method used by the respondents. All (100.0%) the respondents had ever aborted pregnancy more than once and 66.7% reported using contraceptive when pregnancy happened. The mean age of respondents when first abortion was performed was 21.3 ± 3.4. Less than a third (25.9%) of the respondents had ever experienced abortion complications and these were severe bleeding (71.4%) and internal infection of the abdomen (28.6%) (Table 2).

### 3.3. Awareness of HIV Magnitude and HCT

Majority of the respondents were aware of the magnitude of HIV in Nigeria and also considered HIV as a serious health problem amongst sex workers. A respondent in urban brothel specially said

*“I consider myself at risk of sexually transmitted diseases including HIV/AIDS because most times I don't protect myself when having sex because am not always mentally stable because of the drugs I use. Even most times I fight with my clients when I see them because most of them don't pay me because they know am not normal again because of the drugs have injected, so they use the advantage to get a free sex from me and this is always unprotected sex.”*

Few of the respondents were also of the opinion that other women who are not sex workers are also at risk of HIV as sex workers. A respondent in semi-urban brothel said

*“I am at risk of HIV as other women who are not into this business because their husbands come here to do business with us, so if we are infected with HIV then we are going to transfer this to their husband and their husband will also transfer to them at home.”*

Another respondent in urban brothel said

*“I think we are not even at risk of HIV compared with other women who are not sex workers because I can say no to sex if my client refuse to use condom but married women cannot say that to their husband and their husbands are our clients who can easily infect them when infected with this disease from us.” *

On the issue of HIV counseling and testing, some of the respondents reported that they had ever tested for HIV and only one of these respondents disclosed to be HIV positive. Respondents who had never taken HIV test were also asked for their reasons and their reasons were fear of rejection and discrimination by the community and even among other sex workers if the test is positive. Other reasons were inadequate understanding of the importance of HCT, fear of high blood pressure, and depression and shock if tested positive. Many of the respondents reported use of condom, regular talking of herbs, and good personal hygiene as ways of protecting themselves from HIV. A respondent in urban brothel said

*“I have been tested twice this year and the results showed that am negative.”*

Another respondent in urban brothel said

*“I have never been tested because am scared of discrimination even from my colleagues.”*

Another respondent in semiurban brothel said

*“I have not been tested in the last 3 years and I engage in unprotected sex intercourse with many of my clients. Personally, I don't think I need to be tested because if am tested positive I will die of hypertension so I think is better I don't do the test now.”*

### 3.4. Drug Use among Respondents

Cocaine, marijuana, tobacco, and alcohol were reported as the drugs mostly used by the respondents. All the respondents reported sharing needles and injecting equipment. Few of the respondents reported that they were involved in sex work as a means of financing their drug use, while majority reported that they were into sex work before becoming drug users with a major reason to escape from intense burden of their work. Other reasons were to become bold, to be confident to negotiate with clients, and to be strong in bed for clients to enjoy their money. A respondent in urban brothel said

*“I inject cocaine at least twice a week to enable me to be strong and mentally stable. Am actually doing this sex work to raise money to finance the drug am taking.”*

Another respondent in semiurban brothel said

*“I inject drugs many times to enable me to be very strong in bed so that my clients can enjoy me.”*

### 3.5. Condom Use and STI Challenges

All the respondents reported not using a condom at their first intercourse but all had ever used a condom. Less than a third reported using a condom with their last client and more than half reported that ninety percent of their regular clients do not like using a condom. Nearly all the respondents were willing to have clients not to wear a condom in exchange for accepting more money in return, and the majority of them were aware that using a condom could help prevent HIV transmission. Major reason adduced by respondents for inconsistent condom use was mental imbalance effects from drug use. Other reasons were as follows: clients objected to use it, it reduces sexual pleasure, and they did not have one at hand.

A respondent from urban brothel who disclosed to be HIV positive reported to be inconsistent with condom usage with reason that most of her clients always rejected using a condom and even offer more money for this. The respondent specifically said

*“I always try my best to enforce my clients to use condom but majority of them always reject this with reason that condom reduce sexual pleasure and I can't tell them I am HIV positive because of my previous experience.”*

The respondent narrated her previous experience as thus

*“I came from X State to this State to continue this sex work because when I tested positive to HIV, I disclosed this to my friends whom we are doing this business together and within a week the information has spread rapidly to everybody and they started discriminating against me. I suffered a lot during this period as I was not getting any client and life became miserable for me and that was why I travelled down to this State so that I can continue this business as I have nothing else to survive with. Am telling you this because you may assist me with treatment and because you have assured me of my confidentiality and if you don't keep your promise and disclose my status to other sex workers then I will leave this State for another State.” *

The respondent further added that

*“I am actually not seeing any sign on my body to show that I am HIV positive only that I easily get tired and that is one of the reasons for my taking drugs and that has been assisting me a lot.”*

Questions on sexually transmitted infections (STIs) were also discussed with the respondents and more than half of the respondents had ever been treated for STI more than once. Reported STIs among respondents were gonorrhea, syphilis, trichomoniasis, and chlamydia infections. Reported signs and symptoms experienced by respondents were pain during sexual act, pus like discharges from vagina, an itch in the vagina region, and pain during urination. A respondent in urban brothel said

*“I suffered from Gonorrhea last year and it was since then have made up my mind not to allow any client to have sex with me without using condom.”*

Another respondent in semiurban brothel said

*“Have been infected with Gonorrhea and Syphilis in this work and I think it was because of my lack of knowledge of these diseases. Many times when we attend health talk as sex workers it is always HIV/AIDS education so many of us don't really know about other diseases.” *

Another respondent in semiurban brothel said

*“I was told last 2 years that I was infected with Chlamydia infection when I went for medical check-up due to itches in the vagina region which I do experience then and this was treated for me and I was counseled to always use condom with my client when I disclosed to the matron that am a sex worker.”*

## 4. Discussion

The respondents have been in the business for an average of 4.4 years. This is similar to the findings of [15] which also revealed that interviewed FSWs have been in the business for an average of 4.1 years. Majority of the respondents reported having a daily average of 4 clients. In the Russian city of St. Petersburg, female injecting drug users who exchange sex for money had a mean of 49.5 sexual partners in the previous 30 days [16]. All the respondents had ever aborted a pregnancy more than once. This is in accord with the findings of [17] who reported that, in the Netherlands, a large proportion of sex workers, mainly from Latin America and Eastern Europe, do not use contraceptives other than condoms and some have undergone two or three abortions.

All the respondents reported sharing needles and injecting equipment. This is in accord with the findings of [18] where all the respondents reported sharing needles and injecting equipment. The riskiest activity for HIV infection during injection is frequent sharing of injecting equipment with strangers [18]. The highest infection risk lies in the sharing of needles and syringes, where blood is most likely to collect during injection, especially when jacking back (injecting into a vein, drawing blood into the syringe, and reinjecting the blood-and-drug mixture). Even if no blood is visible in either the needle or syringe, HIV may remain trapped in microscopic blood particles. A syringe is a particularly viable atmosphere for HIV within blood, and a contaminated syringe may remain capable of infecting others for up to six weeks [19]. Cocaine, marijuana, and heroin were reported as the drugs mostly used by the respondents and [20] also it is also reported that about one third of the IDUs reported having injected cocaine, heroin, or speedball (heroin and cocaine).

All the respondents reported not using a condom at their first intercourse, but all had ever used a condom. Less than a third reported using a condom with their last client and more than half reported that ninety percent of their regular clients do not like using a condom. Lower levels of condom use have also been shown among those injecting drugs and selling or buying sex in other countries. For example, only 10 percent of injecting drug users from three cities in Indonesia, many of whom had multiple-sex worker and other partners, reported using condom [21]. Among crack using sex workers in Southern Brazil, condom use was reported to be lower with clients who paid more, “looked clean,” and were regulars[22]. Among injecting drug user cohorts in Vancouver, Canada, and in several cities of USA condom use with all types of sexual partners (clients and casual and primary partners) is rare or low among those exchanging sex for drugs or money [23, 24].

More than half of the respondents had ever been treated for STI more than once. This is in accord with the findings of [15] where more than half of the respondents indicated having sexually transmitted diseases once or more. Valdez et al. [20] also reported that 37 percent of respondents reported some history of infection with a sexually transmitted disease since entering sex work. High levels of STIs such as syphilis, gonorrhea, chlamydia, and herpes among sex workers have pointed to the urgent need to provide STI prevention and treatment services for this population [2].

## 5. Conclusion

Respondents have a relatively high number of sexual partners, but this in itself does not necessarily increase their likelihood of becoming infected with HIV; if they use condoms consistently and correctly, then they will probably be protected no matter how many people they have sex with. Some of the respondents reported becoming pregnant even while using family planning and this calls for further investigation to determine the antecedent factors behind this failure. Involving sex workers directly in HIV prevention campaigns can raise their self-esteem and empower them, thereby encouraging them to look after their health and to access services that could help them. Evidence suggests that sex workers who use/inject drugs face increased risk of sexual HIV transmission because they often have a higher number of clients or sexual partners who are more willing to engage in unprotected sex and have sexual partners who also inject drugs [2].

## Figures and Tables

**Table 1:** Demographic characteristics of respondents (*N* = 27).

Demographic variables	*n*	%
Age group		
20–24	6	22.2
25–29	12	44.4
30–34	7	26.0
35–40	2	7.4
Ethnicity		
Igbo	14	51.9
Edo	9	33.3
Yoruba	4	14.8
Marital status		
Single	17	63.0
Separated	8	7.4
Married	2	29.6
Educational level		
Primary education	7	25.9
Secondary education	18	66.7
Tertiary	2	7.4
Years spent in the business		
1–3	4	14.8
4–6	18	66.7
7–10	5	18.5

**Table 2:** Respondents sexual behaviour (*N* = 27).

Sexual behaviour	*N*	%
Age at first intercourse		
13–15	18	66.7
16–19	9	33.3
Feeling at first sexual intercourse		
Pleasure/satisfied	12	44.4
Confused	8	29.6
Cried	7	25.9
Daily average of clients		
3–5	4	14.8
6–10	23	85.2
Currently using family planning		
Yes	24	88.9
No	3	11.1
Type of family planning (*n* = 24)		
Intrauterine device	13	54.2
Condom	9	37.5
Emergency contraceptive	2	8.3
Ever aborted more than once		
Yes	27	100.0
Using contraceptive when pregnancy happened		
Yes	18	66.7
No	9	33.3
Type of contraceptive (*n* = 18)		
Intrauterine device	2	11.1
Condom	9	50.0
Emergency contraceptive	7	38.9
Ever experienced abortion complication		
Yes	7	25.9
No	20	74.1
Complication experienced (*n* = 7)		
Severe bleeding	5	71.4
Internal infection of the abdomen	2	28.6
